# Involvement of main diarrheagenic *Escherichia coli*, with emphasis on enteroaggregative *E. coli*, in severe non-epidemic pediatric diarrhea in a high-income country

**DOI:** 10.1186/s12879-015-0804-4

**Published:** 2015-02-21

**Authors:** Joshua Tobias, Eias Kassem, Uri Rubinstein, Anya Bialik, Sreekanth-Reddy Vutukuru, Armando Navaro, Assaf Rokney, Lea Valinsky, Moshe Ephros, Dani Cohen, Khitam Muhsen

**Affiliations:** University of Gothenburg Vaccine Research Institute (GUVAX), Department of Microbiology and Immunology, The Sahlgrenska Academy of University of Gothenburg, Gothenburg, P.O. Box 435, S-40530 Sweden; Department of Pediatrics, Hillel Yaffe Medical Center, Hadera, Israel; Department of Pediatrics, Laniado Medical Center, Natanya, Israel; Department of Epidemiology and Preventive Medicine, School of Public Health, Sackler Faculty of Medicine, Tel Aviv University, Ramat Aviv, Tel Aviv, Israel; Facultad de Medicina, Universidad Nacional Autónoma de México (UNAM). 3er piso Edificio de Investigación, Circuito interior s/n Ciudad Universitaria, Coyoacán, Mexico; Central Laboratories, Ministry of Health, Jerusalem, Israel; Pediatric Infectious Disease Unit, Carmel Medical Center, Haifa; and Faculty of Medicine, Technion-Israel Institute of Technology, Haifa, Israel

**Keywords:** Diarrheagenic *E. coli*, Enteroaggregative *E. coli*, Sporadic gastroenteritis, Clonal-relatedness, Co-infections, Clinical symptoms, Children

## Abstract

**Background:**

Bacterial and viral enteric pathogens are the leading cause of diarrhea in infants and children. We aimed to identify and characterize the main human diarrheagenic *E. coli* (DEC) in stool samples obtained from children less than 5 years of age, hospitalized for acute gastroenteritis in Israel, and to examine the hypothesis that co-infection with DEC and other enteropathogens is associated with the severity of symptoms.

**Methods:**

Stool specimens obtained from 307 patients were tested by multiplex PCR (mPCR) to identify enteroaggregative *E. coli* (EAEC), enterohemorrhagic (EHEC), enteropathogenic *E. coli* (EPEC) and enterotoxigenic *E. coli* (ETEC). Specimens were also examined for the presence of rotavirus by immunochromatography, and of *Shigella, Salmonella* and *Campylobacter* by stool culture; clinical information was also obtained.

**Results:**

Fifty nine (19%) children tested positive for DEC; EAEC and atypical EPEC were most common, each detected in 27 (46%), followed by ETEC (n = 3; 5%), EHEC and typical EPEC (each in 1 child; 1.5%). Most EAEC isolates were resistant to cephalexin, cefixime, cephalothin and ampicillin, and genotypic characterization of EAEC isolates by O-typing and pulsed-field gel electrophoresis showed possible clonal relatedness among some. The likelihood of having > 10 loose/watery stools on the most severe day of illness was significantly increased among patients with EAEC and rotavirus co-infection compared to children who tested negative for both pathogens: adjusted odds ratio 7.0 (95% CI 1.45-33.71, P = 0.015).

**Conclusion:**

DEC was common in this pediatric population, in a high-income country, and mixed EAEC and rotavirus infection was characterized by especially severe diarrhea.

## Background

Diarrheal disease is a leading cause of pediatric morbidity and mortality in developing countries [1,2], and it is associated with a significant burden in industrialized countries.

The etiology of diarrhea has been studied in both developed [3-5] and developing countries [2,6-8], and it was shown that enteropathogens implicated in the etiology of diarrhea may vary among regions and populations, even when the same epidemiological and microbilogical methods are utilized [2].

Among the major recent advances in molecular diagnosis of enteropathogens is the ability to distinguish between *Escherichia coli* of the normal microbiota and diarrheagenic *E. coli* strains, based on the characterization of virulence genes with polymerase chain reaction (PCR) [9]. Diarrheagenic *E. coli* (DEC) includes different pathotypes of *E. coli* that can induce diarrhea, and are mainly sub-grouped into: enteroaggregative *E. coli* (EAEC), enterohemorrhagic *E. coli* (EHEC) which belongs to the Shiga toxin-producing *E. coli*, typical and atypical enteropathogenic *E. coli* (EPEC) characterized by encoding the bundle-forming pilus (bfp) (typical EPEC), and the enterotoxin-producing enterotoxigenic *E. coli* (ETEC) [9,10]. Despite progress in the field of enteropathogen detection, utilization of PCR-based systems is typically limited to reference laboratories, mostly in the framework of outbreak investigations. Nonetheless, there is public health significance for monitoring and characterization of circulating DEC strains under non-epidemic conditions for rapid identification of emerging virulent strains with potential to cause epidemics such as the recent large-scale epidemic of DEC that occurred in 2011 in Germany, involving more than 3500 cases and 45 deaths [11]. Currently the role of DECs in sporadic pediatric diarrhea in industrialized countries remains under-recognized. Therefore, we aimed to examine the presence of the 4 DEC categories (EAEC, EHEC, EPEC and ETEC) in stool samples obtained from children less than 5 years of age hospitalized for acute diarrhea in Israel and examine the association between mixed infection of DEC with other enteropathogens and clinical manifestation of disease.

## Methods

### Study design and population

A prospective ongoing hospital-based diarrheal disease surveillance network was established in 2007 [12]. The study targeted children less than 5 years of age living in the catchment area of 3 hospitals in Israel. Inclusion criteria included: hospitalization for diarrhea (3 or more watery stools per 24 hours), parents providing an informed consent, and collection of a stool sample. Children were enrolled all year round, and stool samples were obtained from patients within 24 hours of admission. Information about symptoms (e.g., number of loose/watery stools, vomiting, bloody stools, fever ≥39°C) was collected by parental interview and from medical records. Children were excluded from the study if their parents refused to participate. The study included 307 children hospitalized between 2007 and 2011, who had sporadic cases of diarrhea. Since the condition of these children required hospitalization, all were considered as severe diarrhea patients.

The study protocol was approved by the institutional review boards (IRBs) of the Hiller Yaffe, Carmel and Laniado medical centers and by the Ministry of Health. Parents signed written informed consent.

Stool samples (n = 188) were tested for rotavirus antigens by immunochromatography, and for *Shigella*, *Salmonella* and *Campylobacter* by standard stool culture at each hospital's laboratory. A portion of each specimen was sent in cool conditions to the research laboratory at Tel Aviv University where it was frozen at −80°C until plated on MacConkey and CHROMagar ECC plates for detection of *E. coli. E. coli* isolates were then shipped to Gothenburg University in Sweden for identification of the main DEC by mPCR.

### Bacterial growth and preparation of DNA templates

Up to three *E. coli* isolates from each child were cultured for over-night in LB broth at 37°C, and used for preparation of DNA for mPCR. A portion of the over-night culture from each examined bacterial strain was centrifuged and re-suspended by vortex in sterile deionized water. The bacterial suspension was then boiled at 100°C for 5 minutes then centrifuged at a Relative Centrifugal Force of 16,000 × *g* for 2 minutes. Aliquots of the supernatants were frozen at −20°C, and used as template for mPCR.

### Multiplex PCR

The mPCR was developed using specific control *E. coli* strains, or DNA from EHEC, as described previously [13].

Additional microbiological tests were performed on EAEC, which was shown to be involved in diarrhea in other developed country settings.

### Antimicrobial susceptibility

Susceptibility of the EAEC isolates to amoxicillin/clavulanate (AMC), ampicillin (AMP), cefixime (CFM), ceftriaxone (CRO), cefuroxime (CXM), cepahlexin (CL), cephalothin (KF), chloramphenicol (C), ciprofloxacin (CIP), gentamicin (GM), nalidixic acid (NA), norfloxacin (NOR), sulfamethoxazole-trimethoprim (SXT) and tetracycline (T) was determined by the Kirby-Bauer disk diffusion method [14]. The tests were carried out on the Müller-Hinton medium using Oxoid antimicrobial susceptibility disks (Oxoid, Hampshire, England; Becton, Dickinson and Company, Sparks MD, USA), and the interpretations were according to CLSI standards [15].

### O-typing of EAEC isolates

All the identified EAEC isolates were serotyped by agglutination assays using 96-well microtiter plates and rabbit sera (SERUNAM) obtained against 187 somatic antigens and 53 flagellar antigens for *E. coli*, and against 45 somatic antigens for Shigella species [16].

### PFGE analysis

Pulsed-field gel electrophoresis (PFGE) analysis was performed according to the PulseNet standardized PFGE protocol for *E. coli* using *Salmonella* serotype Braenderup strain H9812 as a marker. Agarose-embedded *E. coli* DNA was digested with XbaI (Fermentas) followed by gel electrophoresis in the CHEF MAPPER (Bio-Rad) system. Electrophoresis conditions were 14°C, 0.5×Tris-borate-EDTA buffer, initial pulse 2.2 s, final pulse 54.2 s, 6 V, 18 h. PFGE restriction patterns were analyzed by the BioNumerics software (Applied Maths). Pulsotypes were compared using the band-based DICE similarity coefficient with 1% optimization and tolerance. The un-weighted pair group method with arithmetic mean (UPGMA) algorithm was used for cluster analysis.

### Statistical analysis

The prevalence of DECs and antibiotic resistant strains was presented using frequencies and percentages. The association between detection of enteropathogens and clinical symptoms (having more than 10 loose stools in the most severe day of illness, vomiting, or fever >39°C) was examined using chi square test, and stepwise logistic regression model in which the outcome variable was having more than 10 loose stools on the most severe day of illness (1 = yes, 0 = no), and the explanatory variables in the first step were co-infection between EAEC and rotavirus, *Shigella*, *Salmonella*, and *Campylobacter*. Adjusted odds ratio (OR) and 95% confidence intervals (CIs) were obtained from the model. Two sided Pv < 0.05 was considered significant.

## Results

### Distribution of DEC pathotypes among the *E. coli* isolates

Isolates from 307 children were examined by mPCR, using specific primers as previously described [12], of which 59 (19.2%) were positive for at least one of the four tested DEC. EAEC and atypical EPEC were the most common DEC, each detected in 27 children (46%). ETEC was found in 3 children, while EHEC and typical-EPEC were each found in one child. Co-infection with DEC and another enteric pathogen were common, as revealed by results of stools (n = 188) tested for all DECs, rotavirus and bacterial pathogens. Single infection with DEC was found in 9.5% of children (Table 1).

**Table 1:** **Detection of enteric pathogens in stool specimens of diarrhea patients**

	**No. specimens** ^*^	**%**
Negative to all tested pathogens	55	29.3
Rotavirus only	63	33.5
*Salmonella* only	3	1.6
*Shigella* only	12	6.4
*Campylobacter* only	7	3.7
ETEC only	0	0.0
EHEC only	1	0.5
EAEC only	7	3.7
Atypical EPEC only	9	4.8
Typical EPEC only	1	0.5
Co-infections		
Rotavirus & *Salmonella*	3	1.6
Rotavirus & *Campylobacter*	4	2.1
Rotavirus & ETEC	2	1.1
Rotavirus & EAEC	9	4.8
Rotavirus & atypical EPEC	9	4.8
*Shigella* & EAEC	1	0.5
*Campylobacter* & EAEC	1	0.5
*Campylobacter* & atypical EPEC	1	0.5
Total DEC in both single and co-infections	41	21.8
Total samples tested	188	100.0

^*^This analysis is based on samples from 188 children who were tested for all pathogens presented in the table.

### Clinical symptoms

Information on the various clinical symptoms and enterpathogens was available for 161–191 children. The percentage of children infected with EAEC who had more than 10 stools on the most severe day of illness was significantly higher (50%) than those who were negative for EAEC (19.8%); this percentage was highest in patients with co-infection of EAEC and rotavirus (55.6%), compared to those who were infected with EAEC only (42.9%), rotavirus only (18.8%) or patients who tested negative for both pathogens (21.3%) (Table 2). In logistic regression model, co-infection of rotavirus with EAEC was associated with a 7-fold increased probability of having more than 10 stools on the most severe day of illness (P = 0.015), than children who were negative for both pathogens. Infection with *Shigella* was also strongly associated with having more than 10 stools on the most severe day of illness (Table 3).

**Table 2:** **Clinical symptoms in children with gastroenteritis, by presence of enteropathogens** ^**a**^

	**>10 stools/day n/total (%)**	**Vomiting n/total (%)**	**Fever >39°C n/total (%)**
DEC positive single infection	4/14 (28.6%)	13/16 (81.3%)	9/15 (60.0%)
DEC positive mixed infection	6/22 (27.3%)	18/22 (81.8%)	12/21 (57.1%)
DEC negative	23/121 (19.0%)	113/131 (86.3%)	78/128 (60.9%)
DEC and rotavirus positive	6/21 (28.6%)	19/21 (90.5%)	11/20 (55.5%)
DEC negative and rotavirus positive	14/68 (20.6%)	65/72 (90.3%)	40/71 (56.3%)
DEC positive and rotavirus negative	5/18 (27.8%)	15/20 (75.0%)	12/19 (63.2%)
DEC and rotavirus negative	14/64 (21.9%)	59/71 (83.1%)	40/68 (58.8%)
EAEC positive	8/16 (50%)*	13/18 (72.2%)**	6/17 (35.3%)*
EAEC negative	32/162 (19.8%)	151/173 (87.3%)	102/167 (61.1%)
EAEC and rotavirus positive	5/9 (55.6%)*	8/9 (88.9%)*	2/9 (22.2%)
EAEC negative and rotavirus positive	15/80 (18.8%)	76/84 (90.5%)	49/82 (59.8%)
EAEC positive and rotavirus negative	3/7 (42.9%)	5/9 (55.6%)	4/8 (50.0%)
EAEC and rotavirus negative	16/75 (21.3%)	69/82 (84.1%)	48/79 (60.8%)
Atypical EPEC positive	3/19 (15.8%)	17/19 (85.5%)	14/18 (77.8%)**
Atypical EPEC negative	37/159 (23.3%)	147/172 (85.5%)	94/166 (56.6%)
Atypical EPEC and rotavirus positive	1/10 (10.0%)	9/10 (90.0%)	8/9 (88.9%)
Atypical EPEC negative & rotavirus positive	19/79 (24.1%)	75/86 (90.4%)	43/82 (52.4%)
Atypical EPEC positive & rotavirus negative	2/9 (22.2%)	8/9 (88.9%)	6/9 (66.7%)
Atypical EPEC and rotavirus negative	17/73 (23.3%)	66/82 (80.5%)	46/78 (59.0%)

*Pv <0.05 **Pv <0.1.

^**a**^This analysis in based on the total number of children with complete information on the various clinical symptoms, and detection of enteric pathogens. The total number in each category is indicated in the table.

**Table 3:** **The association between EAEC and rotavirus co-infection with having more than 10 stools in the most severe day of illness**

	Adjusted OR (95% CI)*	**Pv**
EAEC and rotavirus positive	7.00 (1.45-33.71)	0.015
EAEC negative and rotavirus positive	1.56 (0.60-4.07)	0.35
EAEC positive and rotavirus negative	3.84 (0.66-22.2)	0.13
EAEC and rotavirus negative	Reference	
*Shigella* positive	12.49 (2.59-61.15)	0.002
*Shigella* negative	Reference	

*The variables entered in the analysis at step 1 were EAEC/rotavirus, *Shigella*, *Salmonella* and *Campylobacter*. The final model included only EAEC/rotavirus and *Shigella*.

It was also observed that children infected with EAEC were significantly less likely to have high fever (>39°C) compared to EAEC-negative children (35.3% vs. 61.1%) (Table 2). Infection with any DEC (single or mixed infections), was not associated with the above symptoms, neither was atypical EPEC.

### Characterization of the EAEC isolates

Among all 27 EAEC isolates tested for antibiotic resistance, 96% were resistant to CL, 85% CFM and KF, and 78% showed resistance AMP. Lower resistance rates were found to the antibiotics AMC (44.5%), SXT and T (33.5%), and C and NA (18.5%). A low (3–9.5%) resistance rate was found to CIP, CRO and CXM, while no isolate was resistant to GM or NOR.

O-typing of EAEC isolates showed two clusters with the same O and H antigens; O15:H18 and O175:H31. The remaining isolates belonged to different clusters. Although the same O86 antigen was found on two isolates, these differed in their H antigens (Table 4).

**Table 4:** **Distribution of different serotypes among the identified EAEC isolates** ^**a**^

	**Serotype**	
**EAEC isolate**	**O antigen**	**H antigen**
S1	ND^b^	H10
S2	O3	H30
S3	O3	H2
S4	O39	H21
S5	O7	H4
S6	O73	ND
S7	O86	ND
S8	O86	H30
S9	O92	H33
S10	O103	H43
S11	O103	H2
S12	O104	ND
S13	O111	H21
S14	O111	H21
S15	O128	H10
S16	O130	H27
S17	O15	H18
S18	O15	H18
S19	O15	H18
S20	O15	H18
S21	O15	H18
S22	O153	H30
S23	O153	ND
S24	O153	H18
S25	O168	H4
S26	O175	H31
S27	O175	H31

^a^This analysis is based on 27 EAEC isolated that were identified among all 307 samples that were tested for DEC.

^b^Not defined.

**Figure 1:**
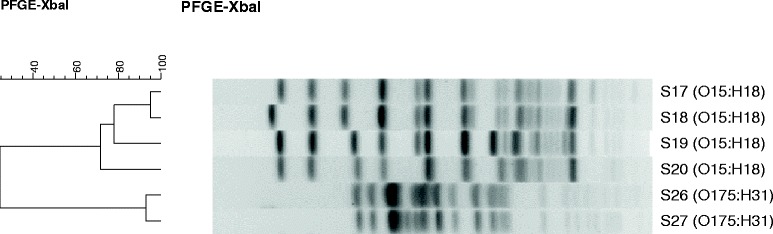
**A dendrogram displaying PFGE profiles of the examined EAEC isolates.**

Pulsed-field gel electrophoresis (PFGE) was applied to assess clonal-relatedness between the isolates having the serotype O15:H18 (isolates S17-S20) or O175:H31 (S26 and S27) (Figure 1). The two isolates within the cluster of O175:H31 were closely related, as there was a difference in one band between the PFGE pulsotypes of these two isolates. Within O15:H18 cluster, the two isolates S17 and S18 were also closely related, while S19 and S20 were different in a few bands and therefore less closely related. The clonal relatedness of the isolates in relation to their PFGE pulsotype patterns was further assessed with the available antibiotic resistance data. Isolates with serotype O175:H31 had a similar but not identical resistance profile to the majority of antibiotics tested (Table 5). A somewhat similar pattern of antibiotic resistance was also seen among the isolates with the serotype O15:H18.

**Table 5:** **Antibiogram of the EAEC isolates with the same serotypes**

**EAEC isolates**	**Serotype**	**AMC 30 μg**	**AMP 10 μg**	**CFM 5 μg**	**CRO 30 μg**	**CXM 30 μg**	**CL 30 μg**	**KF 30 μg**	**C 30 μg**	**CIP 5 μg**	**GM 10 μg**	**NA 30 μg**	**NOR 10 μg**	**SXT 25 μg**	**T 30 μg**
S17	O15:H18	I^a^	R^b^	R	I	S^c^	R	R	R	S	S	S	I	S	S
S18	O15:H18	I	R	I	S	S	S	R	S	S	S	S	S	R	S
S19	O15:H18	I	R	R	I	S	R	R	R	S	S	S	S	R	S
S20	O15:H18	R	R	R	I	S	R	R	S	S	I	S	S	S	S
S26	O175:H31	I	R	I	S	S	R	R	S	S	S	S	S	R	R
S27	O175:H31	I	S	R	S	I	R	R	S	S	S	S	S	S	R

The resistance to antibiotics was examined as described in Methods.

^a^Intermediate.

^b^Resistant.

^c^Sensitive.

## Discussion

A newly developed practical and simple mPCR method [13] was used for the detection of 4 DEC categories (EAEC, ETEC, EPEC and EHEC) in stool specimens from children hospitalized with acute diarrhea. We demonstrated that these 4 DEC pathotypes were common (19.2%) in young children hospitalized with diarrhea in a high-income country. The investigated DECs were found as a sole pathogen in 10% of patients thus indicating that the addition of molecular-diagnosis for DEC identified a substantial portion of enteric pathogens in stool samples of diarrhea patients who tested negative for routinely screened pathogens. EAEC and atypical EPEC were the most prevalent DEC pathotypes. Our findings are in agreement with previous reports from Europe and the United States, showing high prevalence of DEC in patients with sporadic diarrhea [3,5,17].

Notably, EAEC and atypical EPEC comprised the majority of DEC in this study. EAEC is traditionally linked with increased risk of persistent diarrhea [18-21], but it also can cause acute diarrhea among different sub-populations [3,5,21,22]. EPEC is associated with persistent diarrhea in developing countries, and might be implicated in the etiology of diarrhea in industrialized countries, and atypical EPEC is more prevalent than typical EPEC [23-28]. These findings appraise the need for considering antibiotic therapy in DECs-associated acute diarrhea.

A high percentage of EAEC and EPEC infections were mixed with other enteropathogens, mostly with rotavirus. Interestingly, we found that patients co-infected with EAEC and rotavirus had a significant 7-fold increased likelihood of having a large number of watery stools (>10) on the most severe day of illness, compared to children who did not harbor these pathogens. Such an intriguing observation was not found with mixed rotavirus and EPEC infection or with rotavirus and all combined DEC categories. This supports the notion that the observed relationship between mixed rotavirus-EAEC infection and a more severe illness is likely the result of synergy between these two pathogens. One possible explanation is the difference in pathogenesis of illness induced by rotavirus and EAEC. It has been shown that adhesion of different EAEC strains occurs at different locations in the intestine [29], while rotavirus infects enterocytes near the tips of villi of the small intestine [30]. EAEC bacteria adhere to the intestinal mucosa in an aggregative manner forming a characteristic stacked-brick pattern; toxin release then elicits an inflammatory response, mucosal toxicity and intestinal secretion [31]. EAEC toxins can be destructive to the tips and sides of villi and enterocytes [32]. Rotavirus on the other hand primarily causes malabsorptive diarrhea through destruction of absorptive enterocytes and down-regulation of absorptive enzymes [30]. Therefore, we postulate that during EAEC and rotavirus co-infection, both pathogens act simultaneously on the human gut, possibly at different sites, thus resulting in more extensive enteritis, and severe illness. Our unique observation is supported by *in vitro* and animal studies [33] as well as epidemiological studies [34,35] indicating the existence of synergy between rotavirus and *E. coli*, or other pathogens. It is also possible that one infection, either EAEC or rotavirus, creates favorable conditions in the gut for the other infection.

PFGE, O-typing and antibiotic susceptibility pattern were studied in concert with examining clonality of EAEC, which was the dominant DEC in our group of hospitalized children. Using PFGE, genotypic characterization of EAEC isolates of the same serotype showed clusters of isolates having the same pulsotype. In one cluster, two isolates with the serotype O15:H18 showed a difference in only one fragment, which based on generally accepted criteria [36] the isolates would be considered as closely related. Moreover, these isolates had a similar pattern of antibiotic resistance, confirming their close clonal relatedness. Two additional O15:H18 isolates, differing in only a few bands in their pulsotypes, which had a similar pattern of antibiotic resistance, might also be related. The O175:H31 serotype isolates had also similar pulsotypes indicating that these isolates are closely related. Since the children with these isolates were hospitalized in different locations and years, this may suggest that the EAEC strain has been circulating in different regions and time points in Israel.

The multiplex PCR applied in this study had been developed for identification of the main prevalent DEC, i.e. ETEC, EAEC, EHEC, and EPEC. We did not examine the presence of DAEC and EIEC. Therefore, our results may underestimate the prevalence of DEC in acute severe diarrhea in young children. Additionally, the clinical information was lacking in about one third of the patients. However, despite these limitations, our study adds new knowledge regarding the importance of detecting DECs in severe pediatric diarrhea in non-epidemic-conditions in high-income countries.

## Conclusions

With the application of a newly developed practical and simple mPCR method to detect four main DECs categories we demonstrated that EAEC and atypical EPEC are common in children with severe sporadic diarrhea in a high-income country, and that mixed infections with rotavirus and EAEC may influence the severity of the disease.

## Abbreviations

DEC: (diarrheagenic *E. coli*)
MPCR: (Multiplex PCR)
PFGE: (pulsed-field gel electrophoresis)
